# Effect of increasing levels of apparent metabolizable energy on laying hens in barn system

**DOI:** 10.5713/ajas.17.0846

**Published:** 2018-04-12

**Authors:** Hwan Ku Kang, Seong Bok Park, Jin Joo Jeon, Hyun Soo Kim, Ki Tae Park, Sang Ho Kim, Eui Chul Hong, Chan Ho Kim

**Affiliations:** 1 Poultry Research Institute, Rural Development Administration National Institute of Animal Science, Pyeongchang 25342, Korea

**Keywords:** Feed Intake, Intestinal Morphology, Laying Hen Performance, Metabolizable Energy, Nutrient Digestibility

## Abstract

**Objective:**

This experiment was to investigate the effect of increasing levels of apparent metabolizable energy (AME_n_) on the laying performance, egg quality, blood parameters, blood biochemistry, intestinal morphology, and apparent total tract digestibility (ATTD) of energy and nutrients in diets fed to laying hens.

**Methods:**

A total of three-hundred twenty 33-week-old Hy-Line Brown laying hens (*Gallus domesticus*) were evenly assigned to four experimental diets of 2,750, 2,850, 2,950, and 3,050 kcal AME_n_/kg in pens with floors covered with deep litter of rice hulls. There were four replicates of each treatment, each consisting of 20 birds in a pen.

**Results:**

AME_n_ intake was increased (linear, p<0.05) with inclusion level of AME_n_ in diets increased. Feed intake and feed conversion ratio were improved (linear, p<0.01), but hen-day egg production tended to be increased with an increasing level of AME_n_ in diets. During the experiment, leukocyte concentration and blood biochemistry (total cholesterol, triglyceride, glucose, total protein, calcium, asparate aminotransferase, and alanine transferase were not influenced by increasing level of AME_n_ in diets. Gross energy and ether extract were increased (linear, p<0.01) as the inclusion level of AME_n_ in diets increased.

**Conclusion:**

Laying hens fed high AME_n_ diet (i.e., 3,050 kcal/kg in the current experiment) tended to overconsume energy with a positive effect on feed intake, feed conversion ratio, nutrient digestibility, and intestinal morphology but not on egg production and egg mass.

## INTRODUCTION

Dietary energy density, an important nutrient in layer diets, is typically supplied by cereal and protein source and supplemental fat. Dietary energy level can significantly affect the cost of production, because increasing energy levels by the addition of fat can significantly decrease feed intake, increase egg weight, and improve feed conversion [1–3]. Increasing dietary energy by addition of poultry oil has been shown to significantly affect percentage of egg components [4]. There is also a wide range of dietary energy levels (2,684 to 2,992 kcal of metabolizable energy [ME]/kg) currently being used by the egg industry. However, there is limited information concerning the ideal dietary energy level required for optimal laying performance [4]. When the AME content of the diet is increased it could improve nutrient utilization and egg weight [1]. Grobas et al [5] reported that increase in the apparent metabolizable energy (AME_n_) content of the diet from 2,680 to 2,810 kcal/kg decreased feed intake by 5.0% but that egg production and egg mass were not affected. Similarly, Peguri and Coon [6] reported a 5% decrease in feed intake and similar egg production when the AME of the diet was increased from 2,700 to 2,910 kcal/kg. Junqueira et al [7] did not detect any difference in Haugh units or egg shell quality in brown egg laying hens fed diets varying in AME content from 2,850 to 3,050 kcal/kg. The authors have not found any published information on the effects of energy concentration of the diet on serum cotricosterone and intestinal morphology.

The hypothesis of the current experiment was that an in crease in AME_n_ concentration of the diet could improve energy intake and productive performance of the laying hens. To the best of our knowledge, limited research has been published on the increasing levels of AME_n_ on laying performance, leukocyte concentration, blood biochemistry, intestinal morphology, and apparent total tract digestibility (ATTD) of energy and nutrients in diets fed to laying hens in barn system.

## MATERIALS AND METHODS

The protocol for this experiment was reviewed and approved by the Institutional Animal Care and Welfare Committee of the National Institute of Animal Science, Rural Development Administration, Republic of Korea.

### Birds and experimental design

A total of three-hundred twenty 33-week-old Hy-Line Brown laying hens (*Gallus domesticus*) were evenly assigned to four experimental diets of 2,750, 2,850, 2,950, and 3,050 kcal AME_n_/kg in pens with floors covered with deep litter of rice hulls. There were four replicates of each treatment, each consisting of 20 birds in a pen. A commercial type basal diet was formulated to meet or exceed the nutrient recommendations of the National Research Council [8] for layers (Table 1). The diets were analysed in duplicate to determine the crude protein (CP), crude ash, ether extract, and the gross energy (GE) content. Nitrogen for CP analysis was measured using a nitrogen analyzer (NS-2000, Leco Corp., St. Joseph, MI, USA). Dry matter was determined according to the method of Association of Official Analytical Chemists [9], and GE was determined using a Parr adiabatic oxygen bomb calorimeter (Parr Instrument Co., Moline, IL, USA). The diet samples were subjected to analyses of ether extract [9]. The diets were offered daily and were stored at 4°C during the course of the trial. During the 10-week experimental period, hens were provided with feed and water *ad libitum* and were exposed to a 16 h:8 h (light:dark) schedule. The temperature and humidity of the laying house was maintained at 20°C±3°C and 65% to 70% respectively.

### Laying performance

Hen-day egg production rate, floor eggs, broken egg production rate and egg weight were recorded daily, whereas feed intake and the feed conversion ratio were recorded weekly. Egg mass was calculated as per Hayat et al [10].

Egg mass=weekly number of eggs in a replicate×average egg weight

### Determination of egg quality parameters

Ten eggs per replicate were randomly collected at the end of the each week. Eggshell strength, eggshell thickness, egg yolk color, and Haugh units (HU) were measured. Eggshell strength was measured by the Texture Systems Compression Test Cell (model T2100C, Food Technology Co., Ltd., Rockville, MD, USA) and expressed as units of compression force exposed to units of eggshell surface area (kg/cm^2^). Eggshell thickness is defined as the mean value of measurements at 3 different locations on the egg (air cell, equator, and sharp end) and was measured with a dial pipe gauge (model 7360, Mitutoyo Co. Ltd., Kawasaki, Japan) and calculated using the following formula [11]:

Eggshell thickness=(sharp point thickness+equator point thickness+air cell thickness)/3

Egg yolk colour was evaluated by the Roche Yolk Color Fan (Hoffman-La Roche Ltd., Basel, Switzerland; 15 = dark orange; 1 = light pale). Haugh unit values were calculated using a micrometer (model S-8400, Ames, Walthman, MA, USA) with the following formula described by Eisen et al [12]:

$$\text{HU}=100\;\text{log }(\text{H}-1.7{\text{W}}^{0.37}+7.6),$$

Where W is egg weight, and H is albumen height.

### Hematological analysis

At the end of the 10-week feeding trial, 2 birds/replicate (i.e., 8 birds per treatment) with a body weight near the mean were selected to be euthanized by cervical dislocation. Immediately after death, a 5 mL blood sample was collected from the jugular vein of each bird using ethylenediaminetetraacetic acid (EDTA)-treated and non-EDTA treated vacutainer tubes (Becton Dicknson, Franklin Lakes, NJ, USA). The whole blood samples were kept on ice and used immediately for hematological analysis. Leukocytes (white blood cells, heterophils, lymphocytes, monocytes, eosinophils, and basophils) were analyzed using the Hemavet Multi-species Hematology System (Drew Scientific Inc., Oxford, CT, USA). The H/L ratios were determined by dividing the number of heterophils by the number of lymphocytes.

### Blood biochemistry

Serum samples were obtained by centrifuging the samples for 20 min at 25,000×g and 4°C and were stored at −15°C. Total cholesterol, triglyceride, asparate aminotransferase (AST), alanine transferase (ALT), and calcium in the serum were quantified using an ADVIA 1650 Chemistry System (Bayer Diagnostic, Puteaux, France).

### Nutrient digestibility

Birds were fed the experimental diets for 8 d with a 5-d adaptation period to the diet and following 3-d excreta collection period. The marker to marker method was used to ensure total collection of excreta voided from the birds [13]. Chromic oxide (0.3%) and ferric oxide (0.3%) were added to the diet at the start of collection period and at the conclusion of the collection period, respectively. Excreta collection was started when chromic oxide was appeared in excreta, and the collection was finished when ferric oxide was appeared in excreta. Full caution was paid to prevent disturbing the birds whenever the feces were monitored. Feed intake was recorded and excreta were collected on a daily basis, and stored at −4°C be for analysis. Excreta sample were dried in a force-air oven at 60°C for 72 h and finely ground for the subsequent analysis. Diets and excreta samples were analyzed for GE using bomb calorimeter (Parr Instrument, USA). The diets and excreta samples were analyzed for dry matter (Method 934.01), CP (Method 990.93), ether extract (Method 920.39), crude ash (Method 942.05) using standard procedures of Association of Official Analytical Chemists [9]. The ATTD of GE and nutrients in the diet was calculated as: 100 − (total GE or nutrient excretion/total GE or nutrient intake)×100 [14,15].

### Intestinal morphology measurements

Morphometric analysis of the ileum (from Meckel’s diverticulum to the ileo-caeco-colic junction) was evaluated according to Giannenas et al [16]. One-centimetre-long segments were taken from the centre of each part and fixed in 10% buffered formalin for morphometrical assays under light microscopy. Formalin-fixed intestinal tissues were processed, embedded in paraffin wax, sectioned at 3 μm and stained the haematoxylin-eosin method. Histological sections were examined with a Nikon phase contrast microscope coupled with a Microcomp integrated digital imaging analysis system (Nikon Eclipse 80i; Nikon, Tokyo, Japan). Images were viewed using a 4× Eplan objective (40×) to measure morphometric parameters of intestinal architecture. For this purpose, three favourably orientated sections cut perpendicularly from villus enterocytes to the muscularis mucosae were selected from each animal and measurements were carried as follows: villus height was estimated by measuring the vertical distance from the villus tip to villus-crypt junction level for 10 villi per section; crypt depth (the vertical distance from the villus-crypt junction to the lower limit of the crypt) was estimated for 10 corresponding crypts per section.

### Statistical analysis

All data were analyzed by analysis of variance according to completely randomized design using the Proc MIXED procedure of SAS [17]. Outlier data were identified by the UNIVARIATE procedure of SAS, but no outliers were found. The experimental unit for all data was replicate. The LSMEANS procedure was used to calculate mean values. The orthogonal polynomial contrast test was performed to determine linear and quadratic effects of increasing AME_n_ concentrations in diets.

## RESULTS AND DISCUSSION

### Laying performance and egg quality

AME_n_ intake increased (linear, p<0.05) as the inclusion level of AME_n_ in diets increased. Feed intake decreased (linear, p< 0.01) as the inclusion level of AME_n_ in diets increased. Increasing inclusion level of AME_n_ in diets tended to improve hen-day egg production. Hen-day egg production of the 2,850, 2950, and 3,050 kcal/kg treatments compared to the 2,750 kcal/kg treatment trended toward a non-significant increase of 1.09%, 2.54%, and 3.51%, respectively. Feed conversion ratio was improved (linear, p<0.05) with inclusion level of AME_n_ in diets increased. The feed conversion ratio of the other treatments improved by 1.59%, 4.76%, and 7.16%, respectively, compared to the 2,750 kcal/kg treatment (Table 2). Floor eggs, broken egg production, egg weight, and egg mass were not influenced by an increase in the inclusion level of AME_n_ in diets (Table 3).

Hens eat to satisfy their energy requirements, and therefore, an increase in the energy content of the diet should decrease feed intake [18]. However, an increase in the current energy content of the diet from 2,750 to 3,050 kcal of AME_n_/kg (an 11% increase) resulted in a decreased feed intake of 4.7%, resulting in a net increase in energy intake of 5.8%. Bouvarel et al [19] reviewed experiments conducted in laying hens during the last 20 yr and reported that as an average, a 10% increase in AME_n_ content of the diet resulted in a reduction in feed intake of only 5.5%, in agreement with the results of the current experiment. An increase in energy content of the diet is generally attained by increasing the amount of fat and supplemental fat results often in higher energy intake, probably because of less dust formation and improved palatability of the diet [19,20]. Harms et al [2] reported in Brown hens and Single Comb White Leghorns (SCWL) fed diets varying in AME_n_ from 2,500 to 3,100 kcal/kg, there was no significant difference in hen-day egg production with changes in the energy content of the diet. These data support the hypothesis that an excess in energy intake caused by changes in diet composition results primarily in increases in body weight gain rather than in further increases in egg mass production. Egg weight was not affected by energy concentration of the diet, consistent with data of Grobas et al [1] and Bouvarel et al [19]. Bouvarel et al [19] analyzed data from 11 experiments conducted for the last 20 years and reported that egg weight increased 0.96 g per each 10 kcal of extra energy intake per day. The reasons for the discrepancies in relation to the effects of an increase in energy content of the diet on egg weight are not apparent but might depend on the fat and the linoleic acid content of the diet. When the energy concentration of the diet increase, there is usually an increase in both fat and linoleic acid content. Feed conversion ratio improved as the energy content of the diet increased, in agreement with most published reports [1,4,5]. Hens eat feed to satisfy their energy requirements, and therefore, high AME_n_ diets results in improved feed conversion ratio. Moreover, supplemental fat has been shown to reduce rate of feed passage, facilitating the contact between digesta and enzymes and improving digestibility and utilization of other nutrients such as the lipid and carbohydrate fractions of the diet. The results suggest that modern brown-egg-laying hens might not regulate accurately feed intake according to energy requirements when very high or low energy diets are used. Hens fed high AME_n_ diet (i.e., 3,050 kcal/kg in the current experiment) tended to overconsume energy with a positive effect on feed intake and feed conversion ratio but not in egg production and egg mass. Energy concentration of the diet did not affect the percentage of dirty, broken or shell-less egg production rate throughout the laying period, consistent with data of Grobas et al [1].

### Hematological analysis and biochemical blood values

During the experiment, leukocyte concentration and blood biochemistry (total cholesterol, triglyceride, glucose, total protein, calcium, AST, and ALT) were not influenced by increasing level of AME_n_ in diets (Tables 4, 5). Blood parameters are good indicators of general health status, reflecting any physiological, nutritional and pathological changes occurring in the organism; however, it is important to establish appropriate reference values for these parameters [21]. Leukocyte count has also been used as a measure of immune function in birds [22]. Many factors, such as exposure to various microbes and chemicals, can cause changes in both granulocytic white blood cells [23]. The lack of adequate data on the role of increasing level of AME_n_ in altering blood parameters in poultry requires further research.

### Apparent fecal digestibility of nutrients

Nutrient digestibility from the present experiment is summarized in Table 6. Dry matter, CP, crude fiber, and crude ash were not influenced by increasing level of AME_n_ in diets. However, GE and ether extract were increased (linear, p<0.01) with inclusion level of AME_n_ in diets increased (Table 6).

Mateos et al [24] reported that supplemental fat delays the intestinal transit time of the digesta, allowing a better contact between the components of the digesta and the endogenous enzymes present in the gastrointestinal tract, which in turn could improve nutrient utilization. The fat is almost completely absorbed in the small intestine of the bird, and therefore, GE ileal digestibility was expected to be higher for the supplemental fat containing diets [25].

### Intestinal morphology

Intestinal morphology from the present experiment is summarized in Table 7. Villus height increased (linear, p<0.05) as the inclusion level of AME_n_ in diets increased. Crypth depth and villus height:crypth depth ratio was not influenced by increasing level of AME_n_ in diets. Development of intestinal morphology could reflect the health status of the gastrointestinal tract of an animal. New epithelial cells are produced in the intestinal mucosal crypts and migrate along with the villus to the top [26]. As the intestine is the major site of the enzyme digestion and absorption of nutrients, the efficiency of absorption and hence feed conversion ratio largely depends on the morphology of intestine [27]. A deeper crypt may indicate faster tissue turnover to permit renewal of the villus, which suggests that the host’s intestinal response mechanism is trying to compensate for normal sloughing or atrophy of villus due to inflammation from pathogens and their toxins [28]. Previous research has shown that the intestinal morphology, specifically structures such as villus, crypts and the thickness of mucosa, were altered by the composition of diet [29]. The increase in villus height and crypt depth is associated with healthy turnover of epithelial cell and active cell mitosis [30].

## CONCLUSION

Hy-Line Brown laying hens fed high AME_n_ diet (i.e., 3,050 kcal/kg in the current experiment) tended to overconsume energy with a positive effect on feed intake, feed conversion ratio, nutrient digestibility, and intestinal morphology but not in egg production and egg mass.

## Figures and Tables

**Table 1:** Composition and nutrient content of experimental diet

Ingredients (g/kg)	Dietary AME_n_ concentration (kcal/kg)			

	3,050	2,950	2,850	2,750
Corn	529.5	545.2	570.7	596.1
Wheat	50.0	60.0	60.0	60.0
Soybean meal	248.5	242.7	238.0	233.5
Soybean oil	62.5	42.6	21.8	1.0
Mono-dicalcium phosphate	10.8	10.8	10.8	10.6
Limestone	92.7	92.7	92.7	92.8
Sodium chloride	2.5	2.5	2.5	2.5
D,L-methionine-99%	1.5	1.0	1.0	1.0
Vitamin premix1)	1.0	1.0	1.0	1.0
Mineral premix2)	1.0	1.0	1.0	1.0
Total	1,000.0	1,000.0	1,000.0	1,000.0
Calculated composition3)				
AME_n_ (kcal/kg)	3,050.0	2,950.0	2,850.0	2,750.0
Crude protein (g/kg)	175.0	175.0	175.0	175.0
Calcium (g/kg)	38.0	38.0	38.0	38.0
Available P (g/kg)	32.0	32.0	32.0	32.0
Lysine (g/kg)	71.0	71.0	71.0	71.0
Methionine+cyseine (g/kg)	88.0	88.0	88.0	88.0
Analyzed composition4)				
Gross energy (kcal/kg)	3,955.2	3,855.2	3,734.8	3,630.5
Calcium (g/kg)	46.3	45.7	46.0	45.2
DM (g/kg)	903.4	904.6	904.1	901.9
Crude protein (g/kg)	184.4	184.6	184.8	184.7
Crude ash (g/kg)	154.6	154.4	158.4	149.7
Ether extract (g/kg)	55.6	49.9	43.2	27.0

AME_n_, apparent nitrogen corrected metabolizable energy; DM, dry matter.

1) Provided per kilogram of the complete diet: vitamin A (vitamin A acetate), 12,500 IU; vitamin D_3_, 2,500 IU; vitamin E (DL-α-tocopheryl acetate), 20 IU; vitamin K_3_, 2 mg; vitamin B_1_, 2 mg; vitamin B_2_, 5 mg; vitamin B_6_, 3 mg; vitamin B_12_, 18 μg; calcium pantotenate, 8 mg; folic acid, 1 mg; biotin 50 μg; niacin, 24 mg.

2) Provided per kilogram of the complete diet: Fe (FeSO_4_·7H_2_O), 40 mg; Cu (CuSO_4_·H_2_O), 8 mg; Zn (ZnSO_4_·H_2_O), 60 mg; Mn (MnSO_4_·H_2_O) 90 mg; Mg (MgO) as 1,500 mg.

3) Nutrient contents in all diet were calculated.

4) Analyzed in triplicated.

**Table 2:** Influence of the AME_n_ of the diet of the laying hens on productive performance1)

Item	Dietary AME_n_ concentration (kcal/kg)				SEM	p-value	
	
	2,750	2,850	2,950	3,050		Linear	Quadratic
Hen-day egg production (%)	82.7	83.6	84.8	85.6	1.67	0.10	0.28
Floor eggs (%)	2.74	2.77	2.11	1.93	0.75	0.35	0.28
Broken egg production rate (%)	1.76	1.94	1.90	1.16	0.48	0.22	0.36
Egg weight (g)	63.7	63.4	63.0	63.4	0.49	0.85	0.12
Feed intake (g/hen/d)	133.1	131.1	127.9	126.9	1.24	<0.01	0.41
Egg mass (g/d)	52.8	53.0	53.4	54.2	0.93	0.27	0.35
Feed conversion ratio (kg/kg)	2.52	2.48	2.40	2.34	0.05	0.03	0.91
AME intake (kcal/hen/d)	366.1	374.5	377.2	387.3	1.24	0.03	0.35

AME_n_, apparent nitrogen corrected metabolizable energy; SEM, pooled error of mean.

1) Data are least squares means of 4 observations per treatment.

**Table 3:** Influence of the AME_n_ of the diet of the laying hens on egg quality1)

Item	Dietary AME_n_ concentration (kcal/kg)				SEM	p-value	
	
	2,750	2,850	2,950	3,050		Linear	Quadratic
Eggshell strength (kg/cm^2^)	3.96	3.97	3.98	4.06	0.29	0.39	0.35
Eggshell thickness (μm)	374.2	365.6	369.8	376.8	4.59	0.18	0.28
Eggshell color	12.1	12.2	12.2	12.1	0.59	0.79	0.53
Egg yolk color	5.7	5.9	5.6	5.9	0.32	0.35	0.25
Haugh unit	96.3	95.5	95.6	96.4	2.25	0.28	0.37

AME_n_, apparent nitrogen corrected metabolizable energy; SEM, pooled error of mean.

1) Data are least squares means of 40 observations per treatment.

**Table 4:** Influence of the AME_n_ of the diet of the laying hens on blood parameters1)

Item	Dietary AME_n_ concentration (kcal/kg)				SEM	p-value	
	
	2,750	2,850	2,950	3,050		Linear	Quadratic
Leukocytes							
White blood cells (K/μL)	25.45	24.90	25.28	25.48	2.36	0.83	0.83
Heterophils (K/μL)	8.64	8.50	8.38	8.50	0.58	0.71	0.52
Lymphocytes (K/μL)	11.85	12.00	13.17	12.32	1.09	0.67	0.39
Heterophils:Lymphocytes ratio	0.73	0.72	0.65	0.69	0.05	0.89	0.28
Monocytes, (K/μL)	2.53	2.40	2.63	2.38	0.24	0.85	0.15
Eosinophils (K/μL)	1.64	1.74	1.58	1.68	0.29	0.98	0.35
Basophils (K/μL)	0.47	0.58	0.45	0.66	0.55	0.52	0.48

AME_n_, apparent nitrogen corrected metabolizable energy; SEM, pooled error of mean.

1) Data are least squares means of 8 observations per treatment.

**Table 5:** Influence of the AME_n_ of the diet of the laying hens on blood biochemistry1)

Item	Dietary AME_n_ concentration (kcal/kg)				SEM	p-value	
	
	2,750	2,850	2,950	3,050		Linear	Quadratic
Total cholesterol (mg/dL)	125.5	127.7	128.2	129.3	12.03	0.25	0.38
Triglyceride (mg/dL)	1,592.1	1,519.4	1,478.8	1,508.8	110.9	0.36	0.28
Glucose (mg/dL)	191.5	204.7	197.0	200.6	7.30	0.45	0.39
Total protein (mg/dL)	7.00	6.54	5.87	6.87	0.52	0.21	0.53
Calcium (mg/dL)	29.6	28.6	26.1	27.1	1.02	0.52	0.66
AST (U/L)	201.8	233.1	226.6	201.0	7.73	0.22	0.41
ALT (U/L)	1.10	1.21	2.61	1.83	0.31	0.17	0.97

AME_n_, apparent nitrogen corrected metabolizable energy; SEM, pooled error of mean; AST, asparate aminotransferase; ALT, alanine transferase.

1) Data are least squares means of 8 observations per treatment.

**Table 6:** Influence of the AME_n_ of the diet of the laying hens on nutrient digestibility1)

Item	Dietary AME_n_ concentration (kcal/kg)				SEM	p-value	
	
	2,750	2,850	2,950	3,050		Linear	Quadratic
Gross energy (g/kg)	762.0	768.8	805.0	828.0	1.81	0.01	0.66
Dry matter (g/kg)	733.4	703.6	693.0	699.4	2.45	0.32	0.47
Crude protein (g/kg)	567.6	555.8	542.1	591.8	4.02	0.75	0.46
Ether extract (g/kg)	760.0	824.5	868.3	931.8	1.53	<0.01	0.97
Crude fiber (g/kg)	802.9	804.2	810.4	819.2	2.79	0.67	0.89
Crude ash (g/kg)	448.7	422.4	441.6	437.1	3.41	0.92	0.75

AME_n_, apparent nitrogen corrected metabolizable energy; SEM, pooled error of mean.

1) Data are least squares means of 5 observations per treatment.

**Table 7:** Influence of the AME_n_ of the diet of the laying hens on intestinal morphology1)

Item	Dietary AME_n_ concentration (kcal/kg)				SEM	p-value	
	
	2,750	2,850	2,950	3,050		Linear	Quadratic
Villus height (μm)	1,026.3	1,026.8	1,161.3	1,195.8	35.99	0.01	0.65
Crypt depth (μm)	272.0	218.3	272.6	268.3	15.92	0.57	0.15
Villus height:crypth depth ratio	3.92	4.71	4.37	4.50	0.34	0.38	0.35

AME_n_, apparent nitrogen corrected metabolizable energy; SEM, pooled error of mean.

1) Data are least squares means of 5 observations per treatment.
